# EFEMP1 promotes ovarian cancer cell growth, invasion and metastasis via activated the AKT pathway

**DOI:** 10.18632/oncotarget.10296

**Published:** 2016-06-25

**Authors:** Xiuxiu Yin, Shuang Fang, Mei Wang, Qiang Wang, Rui Fang, Jie Chen

**Affiliations:** ^1^ Department of Maternal and Child Health, School of Public Health, Shandong University, Jinan, 250012, China; ^2^ The No.1 People's Hospital of Jining, Jining 272000, China; ^3^ Biochemistry and Molecular Biology, Georgetown University, Georgetown, Washington D.C, 20057, USA; ^4^ Pharmacy Department, Shandong Provincial Hospital affiliated to Shandong University, Jinan, 250012, China; ^5^ Department of Obstetrics and Gynecology, the Second Hospital affiliated to Jilin University, Jilin, 130000, China; ^6^ Clinical Medicine, School of Medicine, Shandong University, Jinan 250012, China

**Keywords:** EFEMP1, ovarian cancer, invasion, metastasis, EMT

## Abstract

EFEMP1, a kind of extracellular matrix (ECM) protein, has been suggested to correlate with the development of different types of carcinoma. However, its functions in ovarian cancer remain unclear. In our study, we performed cDNA microarray analysis and identified EFEMP1 dramatically elevated in the highly invasive subclone, compared with the low invasive subclone. Lentivirus transfection experiments were constructed afterwards. The results demonstrated that knockdown of EFEMP1 significantly inhibited ovarian cancer cell proliferation and induced cell cycle arrest at the G1/G0 phase. We also found that decreased the activity of phospho-AKT could suppress cell invasion and metastasis. Meanwhile, the increased phospho-AKT activity induced by the overexpression of EFEMP1 had significantly enhanced the abilities of ovarian cancer cells to invade and migrate. In addition, the vivo nude mice model confirmed that EFEMP1 was tightly correlated with the development of tumor. The results of RT^2^ Profiler EMT PCR array further indicated that decreased EFEMP1 suppressed epithelial-to-mesenchymal transition (EMT). Collectively, by activating AKT signaling pathway, EFEMP1 contributed to ovarian cancer invasion and metastasis as a positive regulator. Overall, EFEMP1 had showed the potential use in the development of new therapeutic strategies for ovarian cancer.

## INTRODUCTION

Generally, ovarian cancer is accepted as the lethal gynecological malignancy and ranks first in causes of gynecological deaths [1, 2]. The five-year survival rate hovers around 25% ~ 30% and the majority of ovarian cancer patients are diagnosed at advanced stages [3]. Although the substantial advances have been made in cancer therapy, the mortality has remained virtually unchanged over the past 2 decades [4]. Furthermore, tumor recurrence and metastasis are considered as the major reasons for poor clinical outcome and cancer deaths [5]. Therefore, making a thoroughly investigation into the mechanisms of tumor invasion and metastasis may facilitate exploring potential molecular target for ovarian cancer gene therapy.

EFEMP1 (epidermal growth factor–containing fibulin-like extracellular matrix protein 1, fibulin-3) belongs to a multifunctional extracellular matrix protein fibulin family and is critical in maintaining the integrity of the basement membrane and the stability of extracellular matrix (ECM) structure [6–8]. Previous researches have showed that EFEMP1 was correlated with the tumorigenicity of a variety of carcinomas. Song et al. reported the overexpression of EFEMP1 promoted cell proliferation, invasion and adhesion in cervical cancer, which also correlated to the microvascular density and the expression of VEGF [9]. Additionally, compatible results were obtained in the researches of pancreatic adenocarcinoma and glioma [10, 11]. In contrast to the above studies, reports on breast cancer, nasopharyngeal carcinoma and pleomorphic gliomas revealed that EFEMP1 was decreased, and inhibited tumor invasion and metastasis [12–14]. Overall, the function of EFEMP1 in ovarian cancer had not been well illustrated yet.

In previous studies, we found that EFEMP1 expression was increased in ovarian carcinoma compared to normal ovarian tissue, and its overexpression was significantly associated with high stage, low differentiation, lymph node metastasis and poor prognosis of ovarian cancer. EFEMP1 expression was also found over-expressed in the highly invasive subclones compared with the low invasive subclones [15]. Therefore, our intent for this study is to make further investigation of the functional mechanism of EFEMP1 in ovarian cancer cell invasion and metastasis *in vitro*, as well as *in vivo*.

## RESULTS

### Differentially expressed genes in highly and low invasive subclones

**Figure 1:**
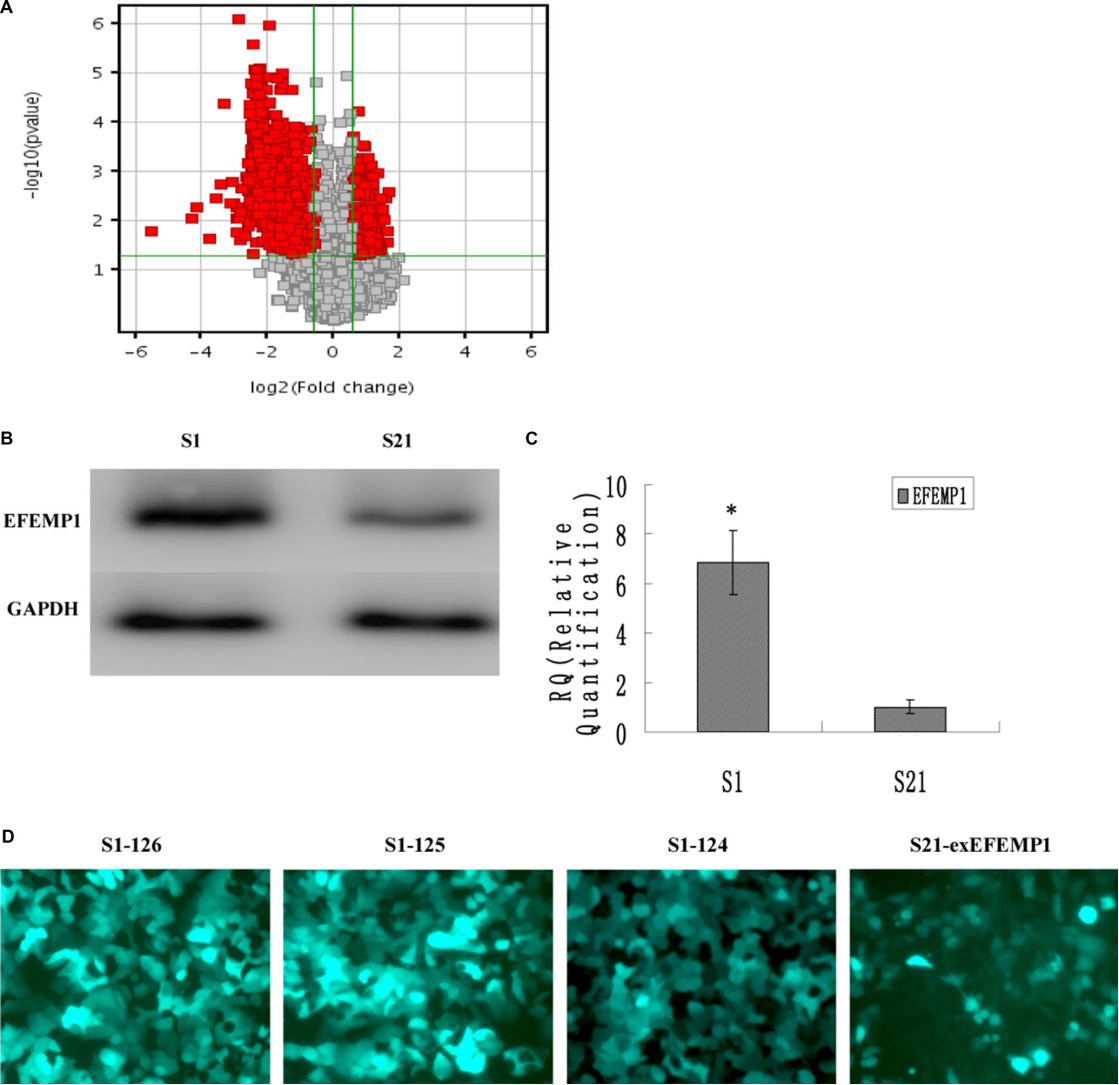
EFEMP1 expression in the highly invasive subclone and the low invasive subclone (**A**) Scatter plot showing fold-change values versus *P*-values for differential gene expression between the highly invasive subclones and the low invasive subclones. The vertical lines correspond to 1.5-fold up and down, respectively, and the horizontal line represents a *p*-value of 0.05. The red point in the plot represents the differentially expressed genes with statistical significance. (**B**, **C**) EFEMP1 expression as measured by Western blot (B) and q-RT-PCR (C). (**D**) The fluorescence images of EFEMP1-GFP (Magnification × 200). *P < 0.05 versus control.

Microarray analysis was performed to identify the differential gene expressions between the highly invasive subclone and the low invasive subclone, which were suitable for comparative analysis because of similar genetic backgrounds. The results revealed 1596 differentially expressed genes of 1.5 fold change and *P*-values ≤ 0.05 (Figure 1A and Supplementary Table S1). Among these genes, compared to the low invasive subclone, EFEMP1 was markedly increased in the highly invasive subclone (11.74 fold, *P* = 0.003). Quantitative real-time PCR and Western blot results confirmed the overexpression of EFEMP1 in the highly invasive subclone (Figure 1B, 1C). In western blot, the average band intensity of EFEMP1 normalized to GAPDH in the highly invasive subclone was 0.85 ± 0.13, much higher than that in the low invasive subclone (0.16 ± 0.04, *P* < 0.05).

### Identification of the efficiencies of EFEMP1 overexpression and knockdown

**Figure 2:**
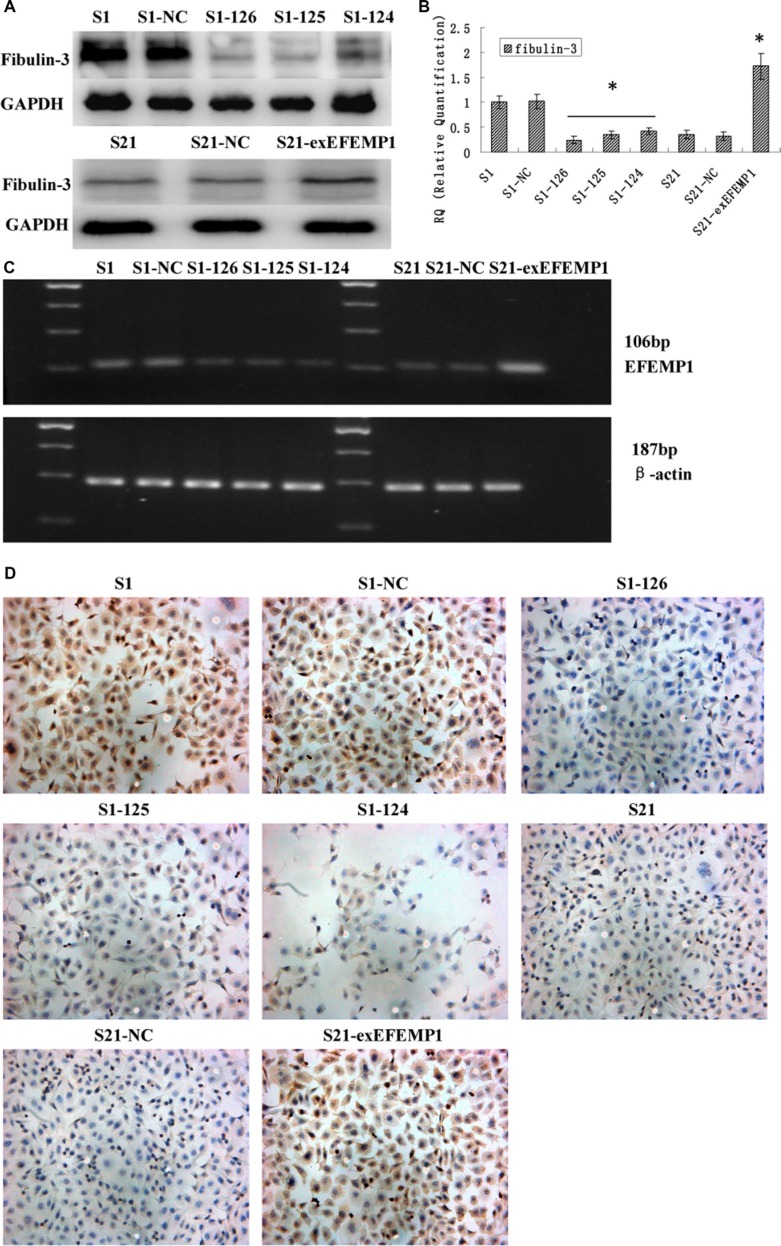
Identification of the efficiencies of EFEMP1 overexpression and knockdown after transfection (S1 and S21: non-infected control, S1-NC and S21-NC: infected with control-shRNA, S1-124: infected with EFEMP1-shRNA 124, S1-125: infected with EFEMP1-shRNA 125, S1-126: infected with EFEMP1-shRNA 126, and S21-exEFEMP1: infected with pLVX- EFEMP1-Puro vector) (**A**) EFEMP1 protein expression in lentivirus-infected cells as measured by Western blot. (**B**) EFEMP1 mRNA expression in lentivirus-infected cells as measured by q-RT-PCR. (**C**) PCR products as measured by agarose gel electrophoresis. (**D**) EFEMP1 protein expression in lentivirus-infected cells as measured by ICC staining (Magnification × 200). *P < 0.05 versus control.

To further investigate the potential role of EFEMP1 in ovarian cancer, we decreased EFEMP1 expression in highly invasive subclone S1, while increased EFEMP1 expression in low invasive subclone S21. After viral infection, more than 80% cells were GFP-positive (Figure 1D), indicating a high efficiency of lentivirus transfection. Real-time q-RT-PCR, Western blot and ICC confirmed the down-regulation and up-regulation of EFEMP1 expressions at both mRNA and protein levels, as shown in Figure 2. In western blot, the average band intensities of S1 and S1-NC normalized to GAPDH in the highly invasive subclone were separately 0.96 ± 0.18 and 0.93 ± 0.19, much higher than that in the EFEMP1 shRNA infected cells S1–126, S1–125 and S1–124 (0.13 ± 0.05, 0.18 ± 0.07 and 0.28 ± 0.06, *P* < 0.05). Otherwise, in the low invasive subclone, the average band intensities of S21 and S21-NC normalized to GAPDH were separately 0.19 ± 0.09 and 0.17 ± 0.08, much lower than that in the pLVX- EFEMP1- Puro infected cells S21-exEFEMP1 (0.68 ± 0.13, *P* < 0.05).

### Effects of EFEMP1 on ovarian cancer cell proliferation

**Figure 3:**
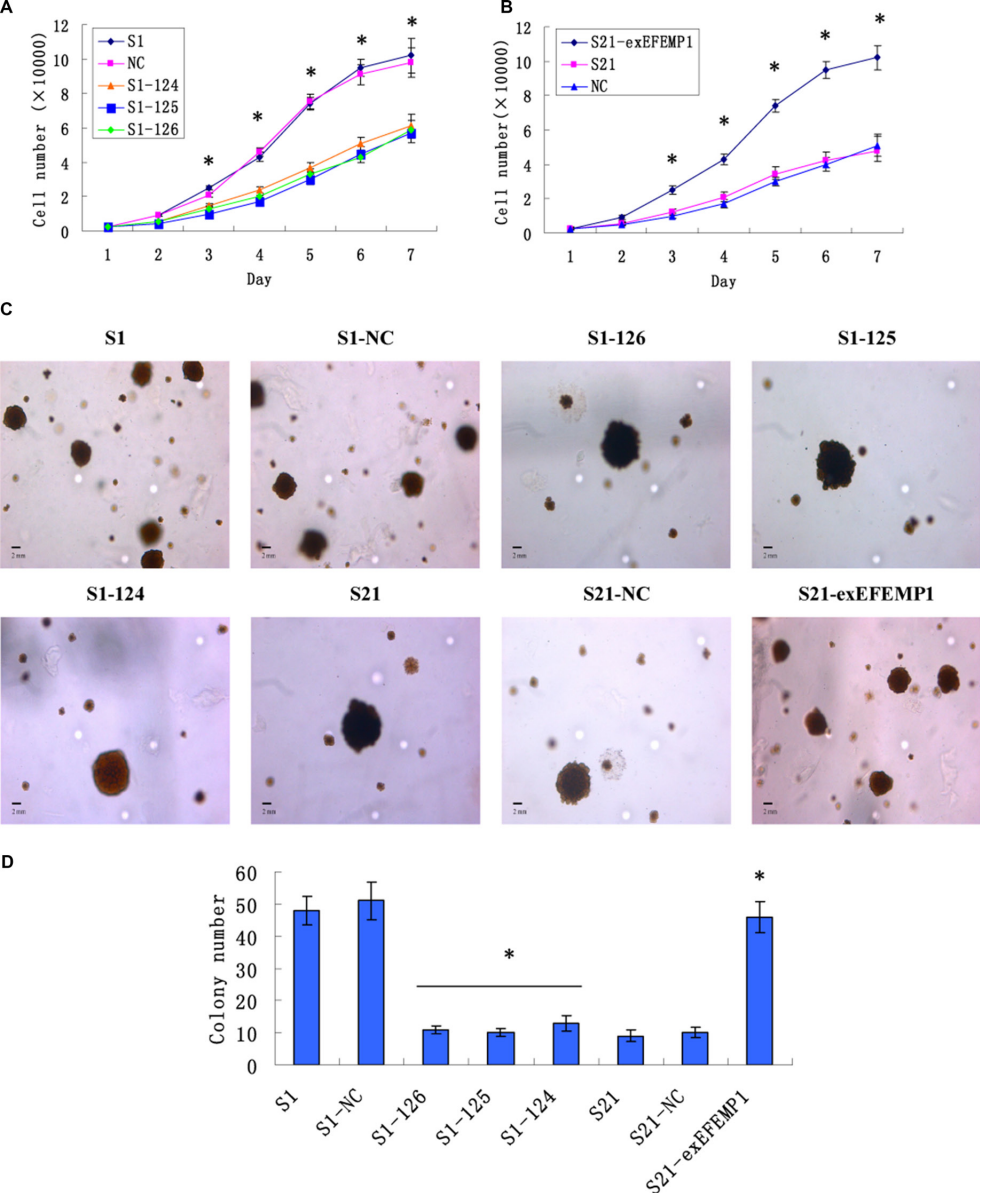
Effects of EFEMP1 on cell growth and cell colony formation (**A**) Knockdown of EFEMP1 inhibited cell proliferation of the highly invasive subclone. (**B**) Overexpression of EFEMP1 promoted cell proliferation of the low invasive subclone. (**C**) The colony images of lentivirus-infected cells as examined by soft agar colony formation assay. (**D**) EFEMP1 knockdown decreased the colony forming capacity of highly invasive subclone, while overexpression of EFEMP1 increased the colony forming capacity of low invasive subclone. *P < 0.05 versus control.

Growth curves revealed that knockdown of EFEMP1 markedly inhibited cell proliferation of the highly invasive subclone (Figure 3A); on the other hand, S21-exEFEMP1 cells with the overexpressed EFEMP1 grew faster than non-infected cells (Figure 3B). In soft agar colony formation assay, the results were consistent with the above. Colony forming efficiency of EFEMP1-silencing cells was decreased, while the exogenous expression of EFEMP1 increased the colony forming capacity of low invasive subclone (Figure 3C, 3D). No significant difference was found in non-infective cells and negative control cells. Due to the heterogeneity of tumor cell, the growth and proliferation abilities of individual cells were relatively different; As a result, the size of the colonies in different groups was quite distinct. The average diameter of colony was also measured, which showed that there was no significant difference between the transfection group and the non-transfection group.

### Effects of EFEMP1 on ovarian cancer cell cycle

**Figure 4:**
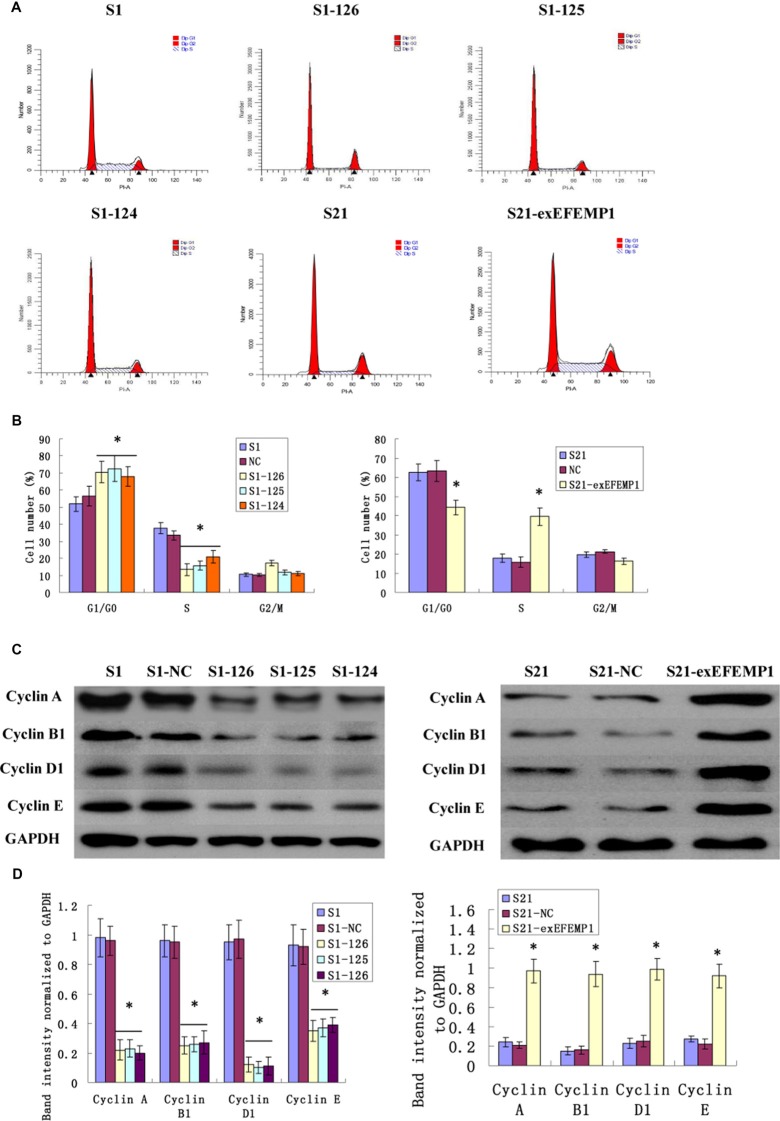
Effects of EFEMP1 on cell cycle (**A**) Cell cycle analysis as measured by flow cytometry in lentivirus-infected cells. (**B**) EFEMP1 knockdown suppressed cell by blocking their progression from the G1/G0 phase to S phase, while EFEMP1 overexpression promoted cell proliferation by increasing the S phase cells. (**C**) Cell cycle-related proteins Cyclin A, Cyclin B1, Cyclin D1 and Cyclin E in lentivirus-infected cells as measured by Western blot. (**D**) The average band intensities of Cyclin A, Cyclin B1, Cyclin D1 and Cyclin E normalized to GAPDH. *P < 0.05 versus control.

Cells infected with EFEMP1 shRNA were exhibited a substantial proportion of cells arrested in the G0/G1 phase compared with negative control, resulting in the sharply decline of cell numbers in S phase. Conversely, the overexpression of EFEMP1 induced a significant decrease in G0/G1 phase and an increase in S phases (Figure 4). These data consistently revealed that silencing EFEMP1 suppressed the proliferation of ovarian cancer cells by blocking their progression from the G1/G0 phase to S phase, while EFEMP1 overexpression promoted cell proliferation by increasing the proportion of cells in proliferate phase (S phase). In other words, EFEMP1 might be an important positive regulator of ovarian cancer cell proliferation. The possible effects on cell cycle-related proteins (such as Cyclin A, Cyclin B, Cyclin D and Cyclin E) during transfection were also considered. Western blot results showed the expressions of Cyclin A (sc-751, Santa Cruz Biotechnology, Inc.), Cyclin B1 (#4138, Cell Signaling Technology, Inc.), Cyclin D1 (#2922, Cell Signaling Technology, Inc.) and Cyclin E (sc-481, Santa Cruz Biotechnology, Inc.) were notably decreased during RNA interference and increased during overexpression transfection (Figure 4C). The average band intensities of Cyclin A, Cyclin B1, Cyclin D1 and Cyclin E normalized to GAPDH in non-infective cells S1 and negative control cells S1-NC of the highly invasive subclones were much higher than that in the EFEMP1 shRNA infected cells S1-126, S1-125 and S1-124. However, in the low invasive subclone, the average band intensities of Cyclin A, Cyclin B1, Cyclin D1 and Cyclin E normalized to GAPDH in non-infective cells S21 and negative control cells S21-NC were much lower than that in the pLVX- EFEMP1- Puro infected cells S21-exEFEMP1 (Figure 4D). Therefore, it was confirmed that EFEMP1 could significantly activate cell cycle regulator proteins and promote cell proliferation in ovarian cancer.

### Effects of EFEMP1 on ovarian cancer cells migration and invasion

**Figure 5:**
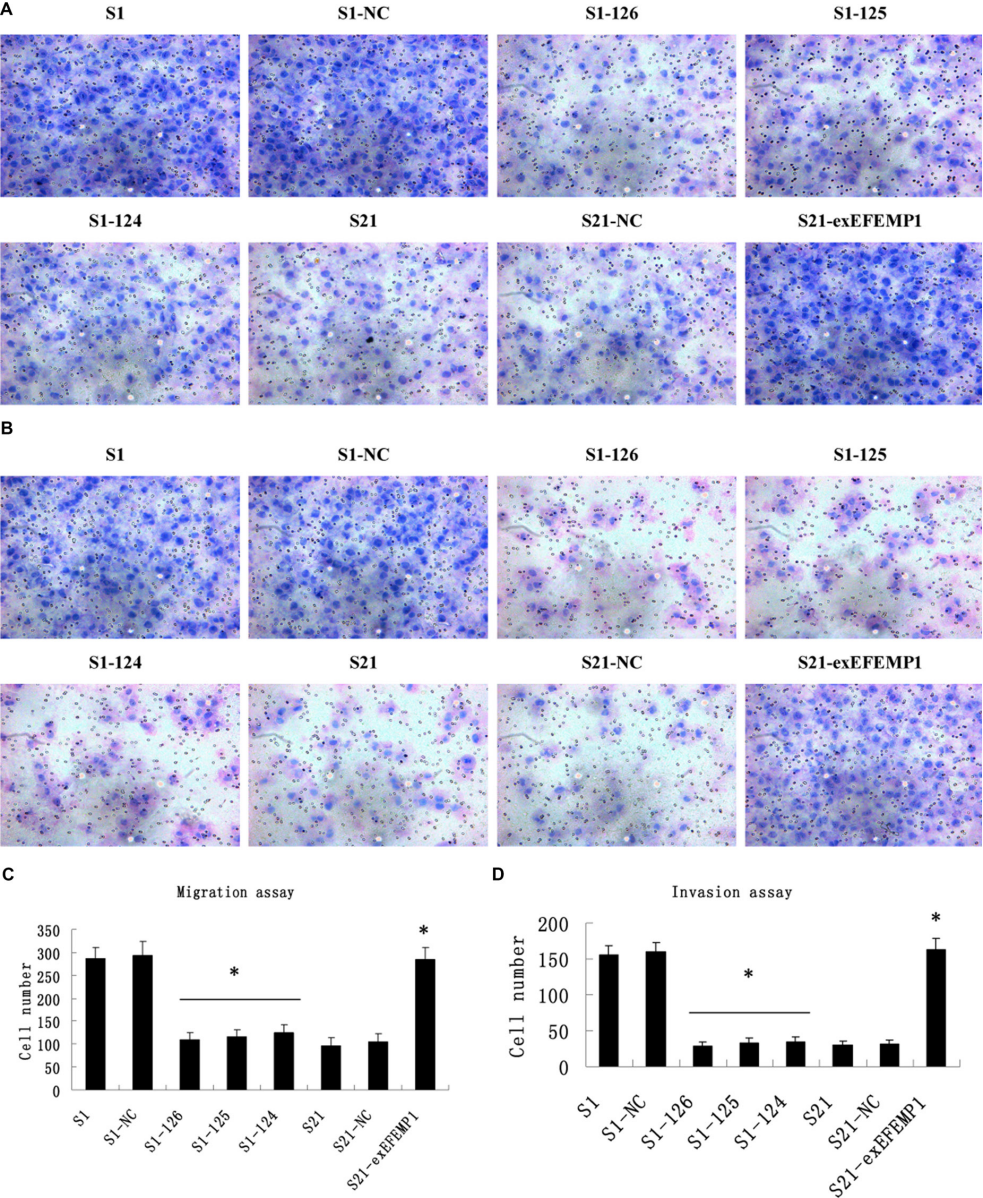
Effects of EFEMP1 on cell migration and invasion (**A**) Cell migration images of lentivirus-infected cells as measured by Boyden chambers without Matrigel. (Magnification × 200). (**B**) Cell invasion images of lentivirus-infected cells by Boyden chambers coated with Matrigel. (Magnification × 200). (**C**) EFEMP1 knockdown suppressed cell migrating abilities, while EFEMP1 overexpression promoted cell migrating abilities. (**D**) EFEMP1 knockdown suppressed cell invading abilities, while EFEMP1 overexpression promoted cell invading abilities. *P < 0.05 versus control.

As shown in Figure 5, the knockdown of EFEMP1 inhibited the invasion and migration of ovarian cancer cells. The average invading or migrating cell count of EFEMP1 shRNA infected cells was much less than that of negative control and non-infected cells. On the other hand, EFEMP1 overexpression promoted ovarian cancer cells invasion and migration. There was significantly more average invading or migrating cell count in S21-exEFEMP1 group, compared to negative control and non-infected groups. No significant difference was found between negative control cells and non-infected cells.

### Effects of EFEMP1 on tumor growth in a xenograft model

**Figure 6:**
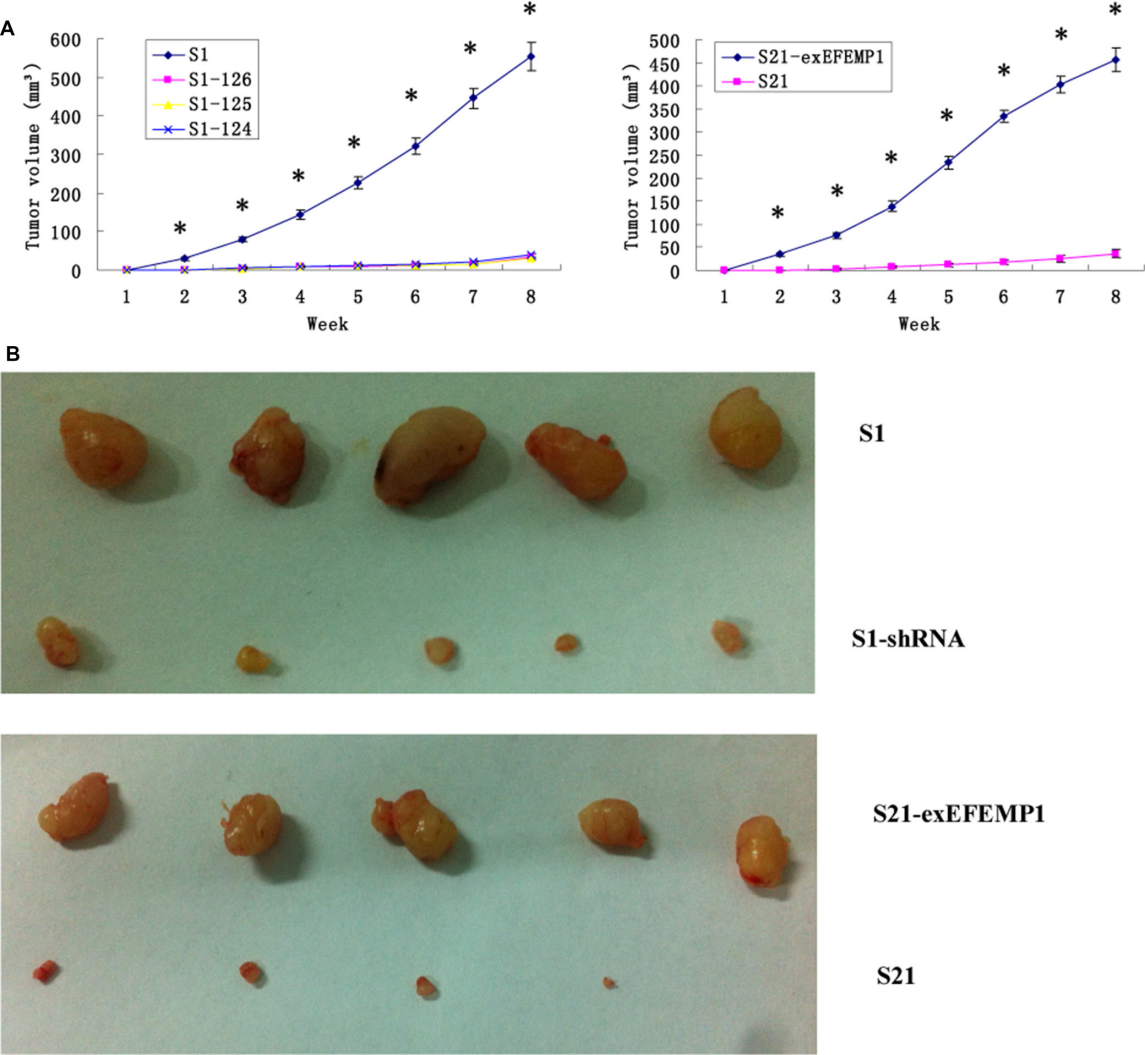
Effects of EFEMP1 on tumor growth (**A**) Tumor growths of lentivirus-infected cells observed continuously for 8 weeks. (**B**) Photograph of xenografts dissected from nude mice after subcutaneous inoculation. *P < 0.05 versus control.

**Figure 7:**
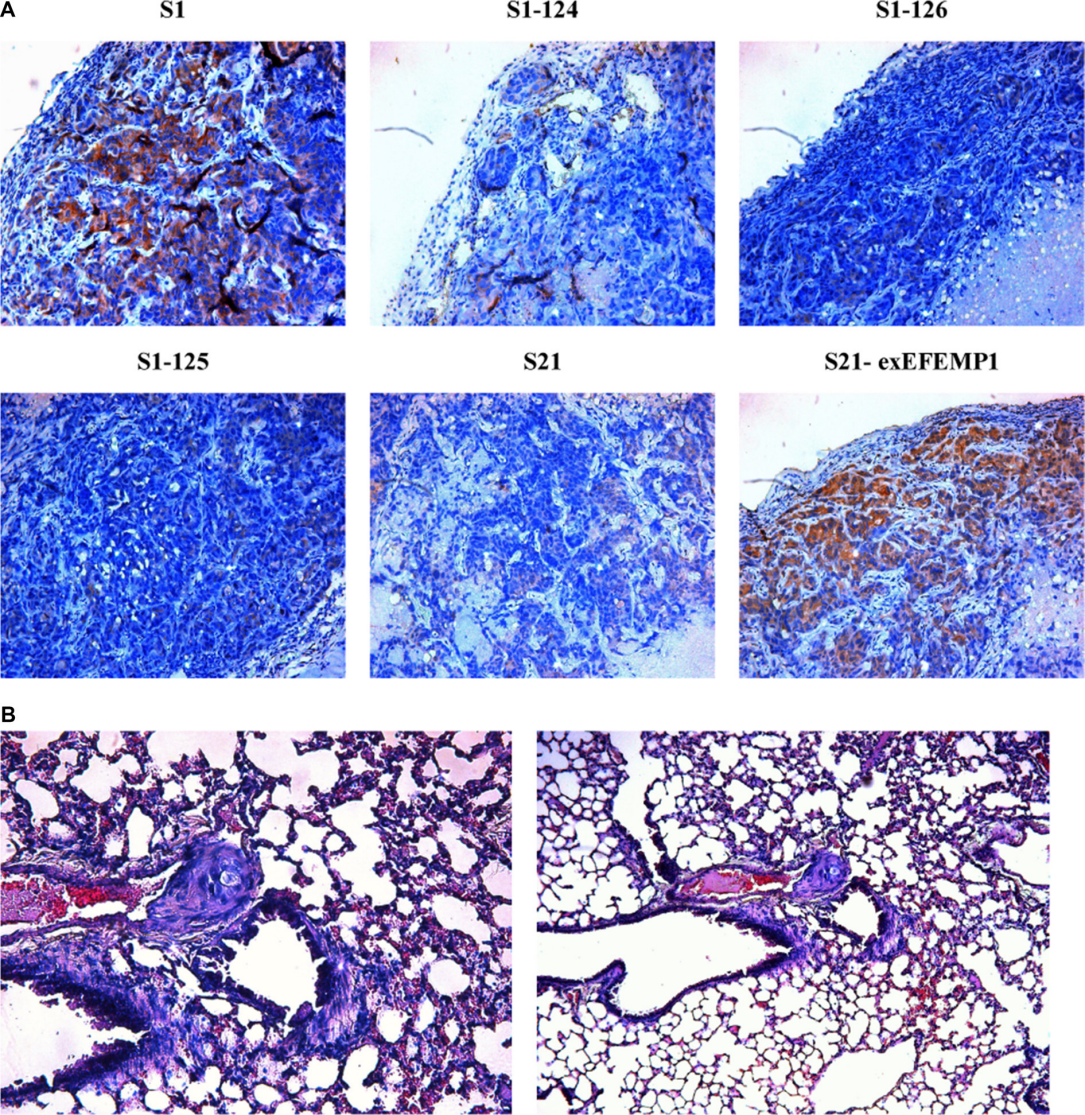
Effects of EFEMP1 on metastasis in nude mice (**A**) Immunohistochemistry were performed on subcutaneous transplantation tumor of lentivirus-infected cells. (**B**) H&E staining were performed on lung metastasis after inoculation through tail vein. (Magnification × 200 and × 100).

To further evaluate the possible function of EFEMP1 in the regulation of tumor growth *in vivo*, we established a xenograft model. S1, S21 and S21-exEFEMP1 were each inoculated subcutaneously and through the tail veins in 5 nude mice respectively, and S1–124, S1–125 and S1–126 were inoculated subcutaneously and through the tail veins in 3 nude mice respectively. The tumor formation rate of highly invasive subclone was as high as 100%, with larger tumor size and rapid growth speed. Meanwhile, there was only 60% tumor formation rate in the EFEMP1-knockdown group, with small size and low growth speed. EFEMP1-knockdown group revealed evident delay in tumor formation and growth. Accordingly, the average tumor size of the nude mice inoculated subcutaneously with S21-exEFEMP1 was larger than that of low invasive subclone group, which had only 80% rate of tumor formation (Figure 6). Collectively, EFEMP1 could promote tumor formation and growth *in vivo*. By immunohistochemistry, the tumor xenografts formed by the high invasive subclones consisted of more EFEMP1 positive tumor tissues, compared to that formed by the EFEMP1 shRNA infected cells (S1–124,S1–125,S1–126), and the S21-exEFEMP1 infected group also has more EFEMP1 positive tumor tissues than the low invasive subclone group (Figure 7A). The EFEMP1-knochdown groups and the low invasive subclone group had no lung metastasis, however, the lung metastasis rates of S1 and S21-exEFEMP1 groups were 100% and 80%, respectively (Figure 7B). In conclusion, EFEMP1 can be a promising booster for ovarian cancer invasive potential.

### Effects of EFEMP1 on the PI3K/AKT pathway

**Figure 8:**
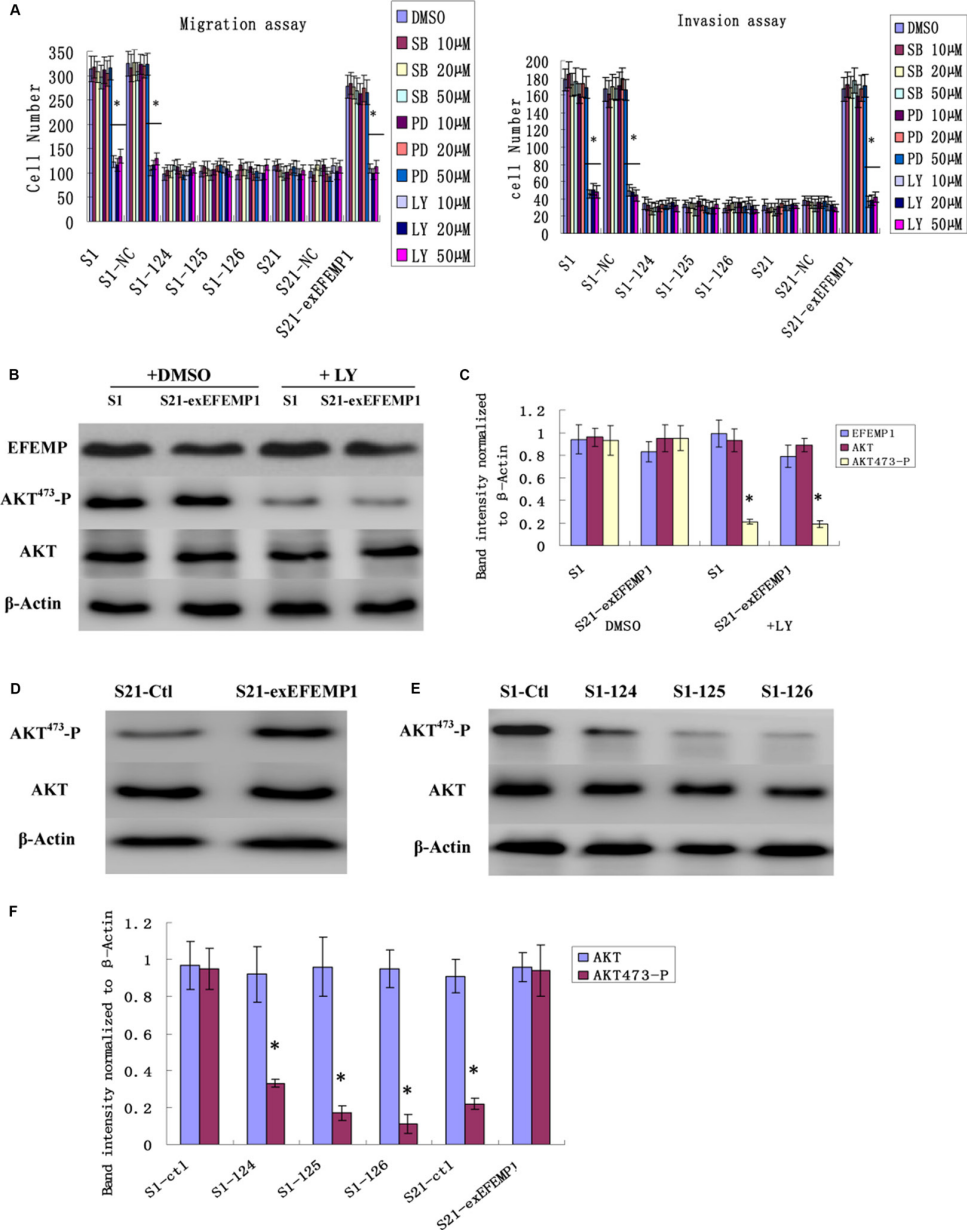
Effects of EFEMP1 on AKT pathway (**A**) EFEMP1 high expressed cells were serum-starved and treated with DMSO or the indicated inhibitors, SB202190, PD98059, and LY294002, for 24 h. The migration and invasion assay were performed by Boyden chambers without or with Matrigel. (**B**) After treating with LY294002, unphosphorylated and phosphorylated forms of AKT were detected by immunoblot analysis. (**C**) The average band intensities were normalized to β-actin. (**D**) After RNA interference, immunoblot analysis were performed to detect the effect of decreased EFEMP1 on the activities of AKT. (**E**) After overexpression transfection, immunoblot analysis were performed to detect the effect of increased EFEMP1 on the activities of AKT. (**F**) The average band intensities were normalized to β-actin. *P < 0.05 versus control.

We studied three signaling pathways (PI3K/AKT, p38, and ERK) using pharmacological pathway inhibitors. EFEMP1 high expressed cells (S1 and the S21-exEFEMP1) were serum-starved and treated with DMSO and the indicated inhibitors, SB202190 (10 μM, 20 μM, 50 μM, p38 inhibitor), PD98059 (10 μM, 20 μM, 50 μM, ERK inhibitor), and LY294002 (10 μM, 20 μM, 50 μM, PI3K/AKT inhibitor), for 24 h. Then, migration and invasion assays were performed. Cell migration and invasion abilities were strongly decreased by incubation with LY294002, but not with SB202190 and PD98059 (Figure 8A). There were no significant differences among the groups of 10 μM, 20 μM and 50 μM, and no dose-dependent pattern was observed. LY294002 significantly reduced the level of AKT-Ser473 phosphorylation and had no effect on the expression of EFEMP1, compared with DMSO control (Figure 8B, 8C). AKT phosphorylation was more diminished in EFEMP1 shRNA infected cells compared with S1 control cells, however in S21-exEFEMP1 infected group, AKT phosphorylation was more increased, compared with S21 control cells (Figure 8D–8F). The results above indicated that EFEMP1 influenced cell migration and invasion involved the PI3K/AKT pathway.

### Effects of EFEMP1 on EMT genes correlated to tumor progression

**Figure 9:**
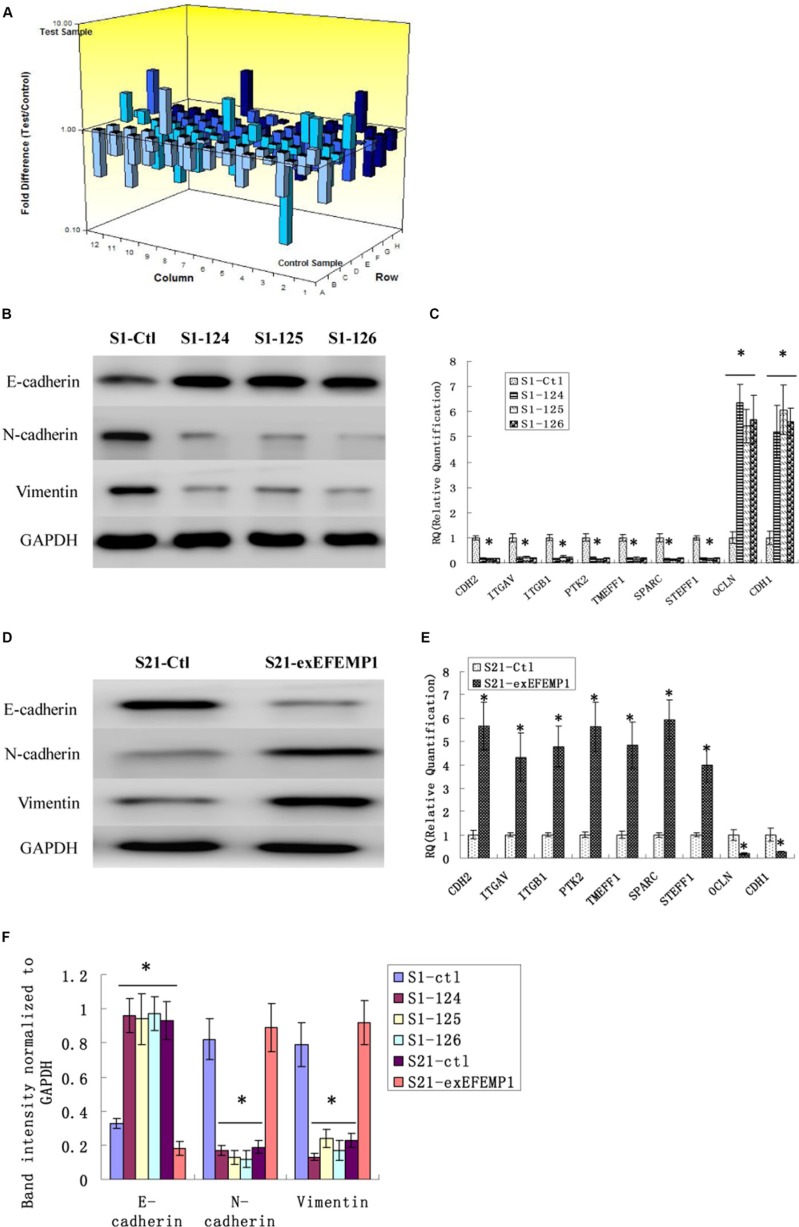
Effects of EFEMP1 on EMT genes correlated to tumor progression (**A**) The microarray image of 84 genes related to tumor progression by RT^2^ Profiler™ PCR Array. (**B**) After RNA interference, EMT marker, including E-cadherin, N-cadherin and vimentin as measured by Western blot. (**C**) The top 9 genes (> 5 folds) of the microarray as measure by real-time qPCR in the EFEMP1 shRNA infected group. (**D**) After overexpression transfection, EMT marker, including E-cadherin, N-cadherin and vimentin as measured by Western blot. (**E**) The top 9 genes (> 5 folds) of the microarray as measure by real-time qPCR in the S21-exEFEMP1 infected group. (**F**) The average band intensities were normalized to GAPDH. *P < 0.05 versus control.

EMT is a key process correlated to tumor invasion and metastasis. Therefore, we examined whether EFEMP1 could stimulate the genes involved in EMT using a focused microarray approach. The EMT microarray contains 84 genes including cell surface receptors; cytoskeletal genes mediating cell adhesion, migration, motility, and morphogenesis. A 2-fold or greater difference in mRNA expression levels was used as the cut-off to determine significant regulatory effects on genes involved in tumor metastasis. It was noteworthy that EFEMP1 had appreciable effect on the expression of several key EMT effectors, including E-cadherin, N-cadherin and vimentin (Table 1 and Figure 9). The down-regulation of EFEMP1 also substantially reduced the expression of a number of genes, including COL5A2, FN1, ITGAV, MMP2, MMP3, MMP9, SMAD2, SNAIL3, TMEFF1, TWIST1 and VCAN, which were increased during EMT (Table 1). We validated the changes seen in the array study by measuring gene expression of the top 9 genes (> 5 folds) using real-time qPCR in the other two shRNA infected cells (S1–124, S1–125), and the S21-exEFEMP1 infected group (Figure 9). The results showed that EFEMP1 down-regulation and up-regulation significantly activated the expression of EMT genes. These results suggest that EFEMP1 has potential to stimulate expression of genes involved in the EMT/metastasis.

**Table 1:** Differential expression of EMT-related genes after EFEMP1 RNA interference

Gene Symbol	Fold Regulation	*p* value
CDH2	−5.32	0.000964
VIM	−4.76	0.012742
COL5A2	−4.44	0.027827
DSC2	−2.51	0.026877
EGFR	−2.37	0.028959
FN1	−2.14	0.000179
FOXC2	−3.80	0.000594
ILK	−4.21	0.001553
ITGAV	−5.40	0.010292
ITGB1	−5.29	0.000010
MAP1B	−2.41	0.014160
MMP2	−4.73	0.033315
MMP3	−4.49	0.006922
PTK2	−5.05	0.000249
PTP4A1	−4.13	0.034422
SMAD2	−3.47	0.001158
SNAIL2	−3.69	0.023954
SPARC	−5.64	0.011191
STEAP1	−5.33	0.000595
TMEFF1	−5.02	0.001031
VCAN	−4.69	0.001605
CDH1	5.61	0.014732
CAV2	4.68	0.021376
FGFBP1	2.65	0.002122
KRT19	3.91	0.004991
KRT7	4.00	0.003723
MST1R	3.01	0.030126
OCLN	5.69	0.018415

## DISCUSSION

In previous study, we confirmed that overexpression of EFEMP1 was closely associated with tumor progression and poor prognosis in human ovarian carcinoma, and EFEMP1 was increased in highly invasive subclone, compared to the low invasive subclone [15]. For further exploring the potential tumorigenicity of EFEMP1 in ovarian cancer, RNA interference and overexpression transfection were made to detect the effects of EFEMP1 on cell proliferation, invasion and metastasis. The influence on EMT genes as well as other possible signaling pathways were also considered.

In particular, EFEMP1 was subjected to both pro-tumor and anti-tumor activities [16]. Our experiment results exhibited that in ovarian cancer, EFEMP1 could promote cancer cell growth, invasion and metastasis via activated the PI3K/AKT pathway. In the HCT116 colon carcinoma cell line, EFEMP1 could promote growth, migration and invasion of cancer cells through a mechanism correlated to p38α and/or p38β activation [17]. In malignant gliomas, EFEMP1 secreted by glioma cells could activate DLL4-Notch signaling to induce pro-angiogenic behavior and promote glioma cell motility and invasion [18, 19]. On the contrary, EFEMP1 inhibited lung cancer cell invasion and metastasis and suppressed MMP-7 expression by activating Wnt/β-catenin signaling [20]. And in nasopharyngeal carcinoma, EFEMP1 suppressed cell migration and invasion via the PI3K/AKT pathway [13]. The role of EFEMP1 has tissue specificity, which may be due to the fact that the functions of tumor-associated genes are determined by the tumor surrounding microenvironment [21].

PI3K/AKT pathway activates numerous cellular functions including cell survival, proliferation, death and invasion, its hyperactivation contributes to tumorigenesis in different cancers [22]. In our study, high-expressed EFEMP1 could increase phospho-AKT activity and promote cells migration and invasion abilities, whereas knockdown its expression could significantly decrease phospho-AKT activity and inhibit cells migration and invasion abilities. In lung cancer stem cells, EFEMP1 could suppress invasion and migration of lung adenocarcinoma cells by modulating the IGF1R/PI3K/AKT/GSK3β pathway [23]. However, in pancreatic carcinoma cells, EFEMP1 could bind to EGFR and contribute to the enhancement of tumor growth via AKT and MAPK signaling pathway [24]. Collectively, EFEMP1 can be one of the regulators of AKT pathway to influence the development of tumor.

As is well known that EMT correlates to the progression of many epithelial cancers, which is closely associated to the dissemination of malignant cells from primary epithelial neoplasm and invasion into the local tissue and blood vessels [25–27]. Therefore, we considered trying to explore the possible relevance between EFEMP1 and EMT in ovarian cancer. Our results revealed that the knockdown of EFEMP1 resulted in increased E-cadherin expression and reduced the expressions of N-cadherin and vimentin; meanwhile, increased EFEMP1 suppressed E-cadherin expression and increased the expressions of N-cadherin and vimentin. EFEMP1 had appreciable effect on the expression of several key EMT genes. In summary, our data simultaneously indicated that EFEMP1 might promote EMT-associated tumor invasion and metastasis in ovarian cancer. In contrary, Kim IG et al. reported that forced expression of EFEMP1 suppressed invasion and migration of lung adenocarcinoma cells and inhibited the EMT process [23]. We speculated that the relationship between EFEMP1 and EMT may be determined by the role of EFEMP1 in different cancers.

The critical conclusion is that our study confirmed that EFEMP1 was a promoting gene for tumor growth, invasion and metastasis in ovarian cancer. Collectively, our findings highlight the importance of EFEMP1 in ovarian cancer development and progression *in vitro* and *in vivo*, which suggests a new therapeutic targeting at EFEMP1 in the future.

## MATERIALS AND METHODS

### Cell line

The highly invasive subclone (S1) and the low invasive subclone (S21) were derived from the SKOV3, human ovarian cancer cell line, which was obtained from Shanghai Institute for Biological Sciences, Chinese Academy of Sciences. The sublones have the highest homology, the smallest heterogeneity and different invasion and metastasis characteristics. Analyzing the gene expression differences between them provides further help for the discovery of genes correlated to the invasion and metastasis in ovarian cancer [28]. Cells were cultured in RPMI-1640 supplemented with 10% fetal bovine serum (FBS) and antibiotics (Gibco BRL, Rockville, MD) at 37°C and 5% CO_2_ condition.

### Microarray analysis

Total RNAs were harvested using TRIzol (Invitrogen) and the RNeasy kit (Qiagen) according to manufacturer's instructions, including a DNase digestion step. After having passed RNA measurement on the Nanodrop ND-1000 and denaturing gel electrophoresis, the samples were amplified and labeled using the Agilent Quick Amp labeling kit and hybridized with Agilent whole genome oligo microarray in Agilent's SureHyb Hybridization Chambers. After hybridization and washing, the processed slides were scanned with the Agilent DNA microarray scanner (G2505B) using settings recommended by Agilent Technologies. The resulting text files extracted from Agilent Feature Extraction Software (version 10.5.1.1) were imported into the Agilent GeneSpring GX software (version 11.0) for further analysis.

### RNA interference and overexpression transfection

The overexpression of EFEMP1 was achieved with Recombining pGC-LV-GV287-GFP vector with the EFEMP1 (NM_001039348) gene, while knockdown of EFEMP1 was achieved with cloning small hairpin RNAs (shRNAs) used self-inactivating lentivirus vector containing a CMV-driven GFP reporter and a U6 (GeneChem, Shanghai, China). The target sequences for EFEMP1 were as follows: 124: 5′- AGTC AATAGTCTACAAATA- 3′; 125: 5′- GTAGACATAGAT GAATGTA -3′; 126: 5′- TGTGAGACAGCAATGCAAA -3′. Specific host cells were incubated with the infection medium containing recombinant vectors at a multiplicity of infection (MOI) of 100 for 24 hours. Then the medium was replaced with fresh complete medium. Ultimately, GFP expression was observed by fluorescence microscopy to inspect the proportion of GFP-positive. The results of western blotting, real-time quantitative RT-PCR and immunocytochemistry (ICC) further confirmed the transfection efficiency.

### Western blot

Protein lysates were separated using 10% SDS-PAGE gel electrophoresis and then transferred to PVDF membrane and blocked with 5% BSA. The primary antibodies EFEMP1 (sc-33722, Santa Cruz Biotechnology, Inc) and GAPDH (sc-365062, Santa Cruz Biotechnology, Inc) were incubated on shaking bed overnight at 4°C. Secondary antibody was incubated at room temperature for 1 h. Developed films were digitized by scanning, and the optical densities were analyzed by the Gel-Pro Analyzer Software (Media Cybernetic, Inc, Bethesda, MD).

### Quantitative real-time PCR (q-RT-PCR) and agarose gel electrophoresis (AGE)

Total RNA was isolated using TRIzol reagent (Invitrogen, CA) and the cDNA was reverse transcribed according to the manufacturers' protocol. Quantitative Real-time PCR analysis was performed in LightCycler^®^ 480 system (Applied Biosystems, USA) with SYBR Green^®^ PCR Master Mix (Applied Biosystems, USA). The 20 μl PCR reaction mixture contained 10 μl SYBR Green PCR master mix, 2 μl cDNA and 0.8 μl the forward and reverse primers each (optimal concentration of 0.2 μM). The primers used for EFEMP1 and b-actin were presented in the following: EFEMP1 sense 5′-ACCCTTCCCACCGTATCCA-3′; and antisense 5′- TCTGCTCTACAGTTGTGCGTCC-3′, β-actin sense 5′-TGGCACCCAGCACAATGAA-3′; and antisense 5′-CTAAGTCATAGTCCGCCTAGAAGCA-3′. Then the PCR products were separated by agarose gel electrophoresis to confirm successful amplification.

### Immunocytochemistry (ICC)

Cells were seeded into 6-well plate with coverslips inside, after appropriate culture, coverslips were subjected to streptavidin-biotin-peroxidase complex procedures (SP). Coverslips were incubated with mouse-anti-human EFEMP1 antibody (sc-33722, Santa Cruz Biotechnology, Inc.) with dilution rate 1:50 at 4°C overnight. The secondary antibody was goat anti-mouse antibody. The positive controls were human ovarian cancer paraffin-embedded sections (EFEMP1 positive), and the negative control was obtained by replacing the primary antibody with normal mouse immunoglobulin (IgG). The presence of brown granules developed by DAB reagent kit in the cytoplasm was considered as positive expression. The EFEMP1 expression was determined by the intensity of staining, quantity of positive cells and distribution.

### Cell proliferation assays

Growth curves and soft agar colony formation assay were used to measure the cell proliferation capacities. For growth curves, logarithmic phase cells were collected and seeded into 24 well plates (1 × 10^4^/well), and further incubated for 7 days. Every day, three wells were taken out to count the number of cells and figured out the average, with growth curves drawn out.

As for soft agar colony formation assay, 1.2% sea plague agar or 0.7% sea plague agar and the same amount of fresh DMEM were used to form base agar layer and top agar layer, respectively. After digested, suspended, cells were seeded into each well. Two weeks later, plates were detected under a microscope to monitor the colony formation. Each well was divided into 4 segments by the horizontal line and vertical line, the numbers of colonies were counted and the diameters of the colonies were measured, then calculated the means.

### Cell cycle analysis by flow cytometry

For cell cycle analysis, cells at log phase were harvested, and washed three times with cold phosphate-buffered saline (PBS). Then stained with 50 μg/ml PI and 250 μg/ml RNase for 30 minutes respectively at room temperature in the dark. The proportion of cells in each phase of cycle was analyzed using the Modfit LT2.0 DNA assay (Becton Dickinson).

### Migration and invasion assays

In vito, transwell assays were performed to assess cell migration and invasion abilities. Briefly, for the migration assays, cells suspended in serum-free media and were seeded into the upper chamber of transwell plate (BD Biosciences, Bedford, MA) with polyvinylpyrrolidone-free polycarbonate (PVPF) filter of 8.0 μm pore size. Conditioned media of NIH3T3 cell culture was added to the lower chamber as a chemotactic factor. After 12 h, the cells on the lower surface of the membrane were fixed, stained with hematoxylin and eosin (H&E) and counted. For the invasion assays, the PVPF membrane of transwell (BD Biosciences, Bedford, MA) was coated with 50 μl Matrigel (BD Biosciences, Bedford, MA) 1:3 diluted with medium, and then cells were seeded in the upper chamber and allowed to invade through the Matrigel to the lower chamber for 24 h. Then non-invading cells remaining on the upper surface were removed, the number of invaded cells (lower side of the membrane) were fixed, stained and counted. Each experiment was performed at least in triplicate.

### Tumor xenografts in nude mice

BALB/C-nu/nu nude mice (female, 5-week-old) were purchased from National Resource Center for Rodent Laboratory Animal of China for tumorigenicity assays. Nude mice were injected subcutaneously or through the tail veins with 5.0 × 10^6^ fresh cells. All mice were raised in the same sterile animal facility. After regularly monitored for 8 weeks, the mice were killed and the lung and tumor tissues were made into paraffin-embedded sections for immunohistochemical examination. Tumor onsets were measured with calipers weekly by two trained laboratory staffs at different times on the same day. The volumes of tumors were calculated according to the formula: (length × width^2^)/2 and the average values were expressed as mean ± SE. The animal experiment was approved by the Institutional Animal Care and Use Committee of Shandong University and was in compliance with all regulatory guidelines.

### RT^2^ profiler™ PCR array

In order to detect the effect of EFEMP1 on the gene expression of epithelial mesenchymal transition, the Human Epithelial to Mesenchymal Transition (EMT) RT^2^ Profiler™ PCR Array (PAHS-090, SABiosciences) were performed by KangChen Bio-tech (Shanghai, China). After RNA isolation, DNase treatment and RNA clean-up, the isolated RNA was reverse transcribed into cDNA according to the manufacturer's protocol. Real-time PCR Array was done using Super Array PCR master mix on an ABI PRISM7900 instrument (Applied Biosystems, Foster City, CA). Data were analyzed using ΔΔCt method.

### Statistical analysis

The results of cell and molecular biology data were expressed as the mean ± SE. For analysis of statistical significance between two preselected groups, a two-tailed *t*-test was used, and for analysis of statistical significance among three preselected groups, one-way ANOVA was used. Statistical analysis was performed using SPSS statistical software package (standard version 13.0). *P*-value < 0.05 was considered statistically significant.

## SUPPLEMENTARY MATERIALS




