# Supplementary material for: EFEMP1 promotes ovarian cancer cell growth, invasion and metastasis via activated the AKT pathway
Source: Oncotarget. 2016 Jun 25;7(30):47938–53. doi: 10.18632/oncotarget.10296 (PMC5216990; doi:10.18632/oncotarget.10296)
Supplement: Supplementary file 1 [file oncotarget-07-47938-s001.pdf]

EFEMP1 promotes ovarian cancer cell growth, invasion and metastasis via activated the AKT pathway

Supplementary Materials

Supplementary Table S1: Differentially expressed genes (low invasive cell vs highly invasive cell)
Column A: ProbeName; Column B: P-value calculated from TTest; Column C: Fold change, the ratio of normalized intensities between two conditions; Column D ~ F: log2 Ratio of two channels. Column G ~ P: annotations to each probe, including GenbankAccession, Map, GeneSymbol, Description, GO annotations, RefSeq, Unigene, Dbid. See Supplementary_Table_S1
